# Causal Association Between Oral Microbiota and Major Salivary Gland Cancer: A Bidirectional Two-Sample Mendelian Randomization Study

**DOI:** 10.3290/j.ohpd.c_2333

**Published:** 2025-11-12

**Authors:** Yanting Ip, Hoilun Chu

**Affiliations:** a Yanting Ip MS Student, School of Stomatology Jinan University, China. Conceptualization, methodology, supervision, wrote, edited and reviewed the manuscript, project administration, funding acquisition.; b Hoilun Chu Undergraduate Student, Professional affiliation: School of Stomatology Jinan University, China. Methodology, investigation, data curation, wrote the orignial draft, visualization.

**Keywords:** causality, major salivary gland cancer, Mendelian randomization, oral microbiota.

## Abstract

**Purpose:**

The two-sample Mendelian randomization approach was used to assess the potential causal relationships between 33 oral microbes and salivary gland cancer.

**Materials and Methods:**

The data used in this study were obtained from summary statistics of genome-wide association studies (GWAS). The instrumental variables included 33 known oral microbes, comprising a total of 39,117,105 single nucleotide polymorphisms (SNPs). The outcome variable, major salivary gland cancer (MSGC), included 11,831,294 SNPs. Mendelian randomization (MR) analysis was conducted via inverse-variance weighted (IVW) as the primary method. To ensure the robustness of the results, Cochran’s Q test, the MR-Egger intercept test, leave-one-out analysis, and reverse MR analysis were employed.

**Results:**

The IVW analysis results indicated that the genus *Alloprevotella* (odds ratio [OR] = 1.267; 95% confidence interval (CI) = 1.037–1.549; p = 0.020) and the species *Veillonella dispar* (OR = 1.369; 95%CI = 1.081–1.734; p = 0.009) are statistically significant risk factors for the occurrence of MSGC.

**Conclusion:**

The genus *Alloprevotella* and the species *Veillonella dispar* have a positive causal relationship with major salivary gland cancer.

Major salivary gland cancer (MSGC) is a type of head and neck cancer, accounting for approximately 3% of all head and neck tumors. Owing to its morphological diversity and overlapping features, malignant MSGC is considered one of the most challenging areas in pathological diagnosis.^23,37^ According to the WHO Global Cancer Observatory, 53,583 new cases of MSGC were diagnosed worldwide in 2020, 43% of which occurred in elderly individuals, leading to 12,339 cancer-specific deaths. As the aging population continues to grow, the incidence of MSGC is expected to rise, posing significant challenges to clinical diagnosis.^4,5,38^ Previous studies have suggested that most MSGCs are caused by inflammation. These cancers primarily occur in the parotid gland and manifest in various pathological forms, such as mucoepidermoid carcinoma.^36^ Additionally, research has shown that MSGCs have a high tendency for lymphatic metastasis.^40^ However, the exact causes of MSGC remain unclear, and understanding the risk factors for salivary gland cancer is crucial for public health and disease prevention.

Previous studies have shown that bacteria are closely associated with the development and progression of tumors.^27,43^ In recent years, microbes in the oral cavity have been shown to be significantly related to tumors.^1,50^ The human oral cavity is typically colonized by nearly 1000 different microbial species and their subsets, making the oral microbiota one of the most essential and complex microbial ecosystems in the human body.^16^ Although the oral microbiota has long been considered relevant only to oral diseases such as caries and periodontal disease, as early as 1891, oral microbiologist Willoughby D. Miller suggested that oral microbial infections could impact systemic health beyond the oral cavity.^26^ Recent studies have revealed a statistically significant correlation between the abundance of specific microbial species in the salivary microbiome and the occurrence of extraoral cancers, indicating that these findings may reveal potential environmental factors associated with cancer development.^2^ Studies suggest that the mucosal microbiota can become part of the tumor microenvironment in respiratory and gastrointestinal malignancies, influencing cancer growth and spread through various mechanisms. These mechanisms can be categorized into three main pathways: (i) altering the balance between host cell proliferation and apoptosis, (ii) modulating immune system functions, and (iii) affecting the metabolism of host factors, ingested food, and drugs.^9^ Therefore, it is reasonable to speculate that oral cavity microbes can also influence the development of MSGC. To determine the causal relationship between oral cavity microbes and MSGC, we conducted a study via Mendelian randomization (MR) analysis. This method requires the use of exposure factors significantly associated with oral cavity microbes as instrumental variables (IVs).

Demmitt’s study^6^ showed that the oral microbiome diversity of monozygotic twins is significantly lower than that of dizygotic twins or unrelated individuals and that this phenomenon is independent of cohabitation status. The result of 752 twin pairs modeling shows that most microbial traits exhibit greater than 50% heritability, indicating that host genes play a significant role in shaping the microbiome.^6^ Specific host genes that interact with the oral microbiome are believed to be the primary source of this heritability. In response to this phenomenon, the mainstream concept of infectogenomics in academia is that there are genetic defects in the host’s recognition and response pathways to microbial pathogens, which can lead to changes in microbial colonization or misrecognition of normal microbiota, thereby causing dysbiosis and the emergence of infectious diseases.^14,28^ In this case,the best method for identifying these genes is to analyze the associations between single nucleotide polymorphisms (SNPs) and traits.^10^ In other, similar studies, the influence of host genetic factors on the oral microbiome has also been confirmed.^7^ Therefore, SNPs are employed as IVs in the present study. This study aimed to investigate the correlation between oral cavity microbes and MSGC via a bidirectional two-sample Mendelian randomization approach based on genome-wide association studies (GWAS) databases, providing a foundation for identifying biological factors associated with major salivary gland tumors.

## MATERIALS AND METHODS 

### Mendelian Randomization Research Design

Mendelian randomization is a crucial research method in epidemiology and is widely used to explore potential causal relationships between exposure factors and clinical diseases. Past studies leverage SNPs from GWAS as instrumental variables in measuring exposures. Owing to the random allocation of individual alleles, which are typically innate factors, this approach effectively reduces endogeneity issues between exposures and disease. Among the different MR methods, past research has suggested that two-sample Mendelian randomization (TSMR) offers considerable advantages in reducing uncertainty in causal estimates and minimizing confounding bias, making it more efficient for exploring causal relationships between diseases and related factors.^17^ Hence, this research engages the TSMR, which uses SNPs as IVs to measure the categories of the oral microbiota, and explores its causal relationship with the MSGC.

**Fig 1:**
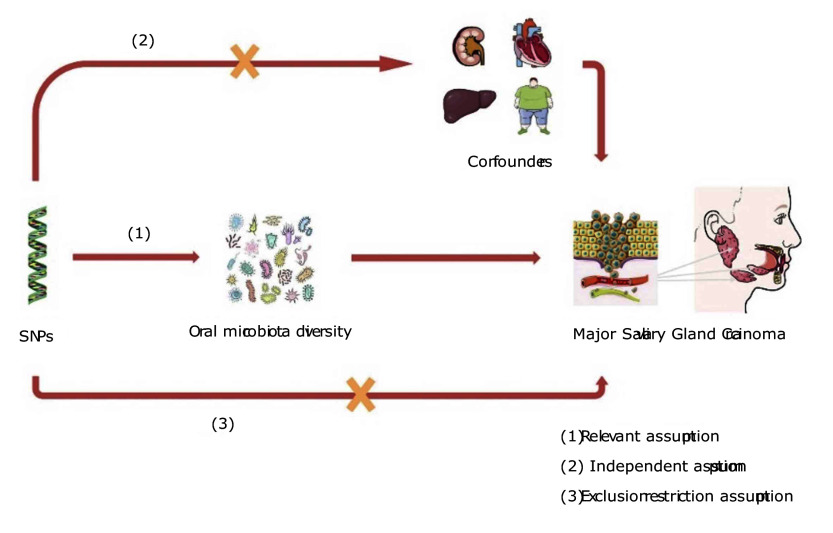
The principal assumptions and design of Mendelian randomization analysis.

To ensure the accuracy and validity of the bidirectional TSMR results, this study design adheres to three key assumptions: (1) relevance assumption: the selected IVs must have a strong correlation with the oral microbiome; (2) independence assumption: the IVs must independently affect only the oral microbiome as the exposure factor and should not influence other traits; (3) exclusivity assumption: the IVs influence MSGC occurrence solely through their impact on the oral microbiome and must not directly affect the MSGC. The detailed workflow is shown in Fig 1.

### GWAS Data Sources

The summary-level statistics for the oral microbiota and MSGC were obtained from previous studies or consortia. All studies were approved by their respective institutional review boards, and no new institutional review board approval was needed.

Our exposure factor, the oral microbiota, was assessed from Stankevic’s study^39^ in the GWAS catalog. Their research included 610 individuals from the ADDITION-PRO cohort in Denmark and 43 oral microbes species. Excluding 10 unknown species, 33 oral microbes species and their corresponding 39,117,105 SNPs were included in this study.

The outcome variable, MSGC, was collected from the study by Jiang et al^11^ in the GWAS catalog. Their research utilized 456,348 European individuals, including 105 samples in the treatment group and 456,243 samples in the control group, from the UK Biobank (full summary statistics available at http://fastgwa.info/ukbimpbin) to conduct a secondary analysis, examining the relationships between 11,842,647 SNPs and MSGC via a generalized linear mixed model.

### IVs Selection

To ensure the validity and accuracy of the TSMR, this study systematically screened the SNPs associated with 33 known bacterial species in the oral microbiota dataset. The steps are as follows: (1) Due to the scarcity of remaining SNPs under the p < 5×10^8^ statistical significance threshold, the statistical significance threshold was set to p < 5x10^5^ in the SNP-oral microbiota and p < 5x10^6^ in the SNP-MSGC. The strength of IVs was assessed with the F-statistic, which is calculated via the following formula:

F = (β/SE)^2^

When the corresponding F-statistic is >10, it can be concluded that there is no statistically significant weak instrumental bias and that the outcome is reliable.^18^ (2) Since potential pleiotropy among SNPs may cause linkage disequilibrium (LD) and lead to biased results, the SNPs were excluded according to the LD threshold, with r^2^ < 0.001 and a clumping window of 10,000 kb to mitigate the impact of gene pleiotropy on the outcome.

### Statistical Methods

This study utilized R software (Version 4.3.3) and the Two Sample MR package (Version 0.6.7) to perform MR analysis on SNPs associated with 33 selected oral microbes. The inverse-variance weighted (IVW) method is the primary method for evaluating causal effects.^20^ To mitigate the concern of susceptibility to pleiotropy bias, further tests were employed, such as the Cochran Q test, MR-Egger method, MR-PRESSO method, and leave-one-out analysis, to assess the reliability of the results. The Cochran Q test is used to determine whether heterogeneity exists among the selected IVs. The MR-Egger method evaluates the presence of horizontal pleiotropy by referencing the intercept term; when the intercept is close to zero, it indicates that MSGC occurrence has no specific tendency. The MR-PRESSO technique identifies and corrects outlier SNPs among the IVs, enhancing the reliability of causal effect estimation. Finally, the leave-one-out analysis systematically excludes individual SNPs one at a time to calculate the combined effect of the remaining SNPs, ensuring that the outcome is not disproportionately influenced by any single SNP. Finally, to address the potential bidirectional endogeneity between the oral microbiota and MSGC, the places of the exposure variable and the outcome variable were exchanged to clarify the causal relationship.

## RESULTS

### IV Selection and MR Analysis Results

**Fig 2:**
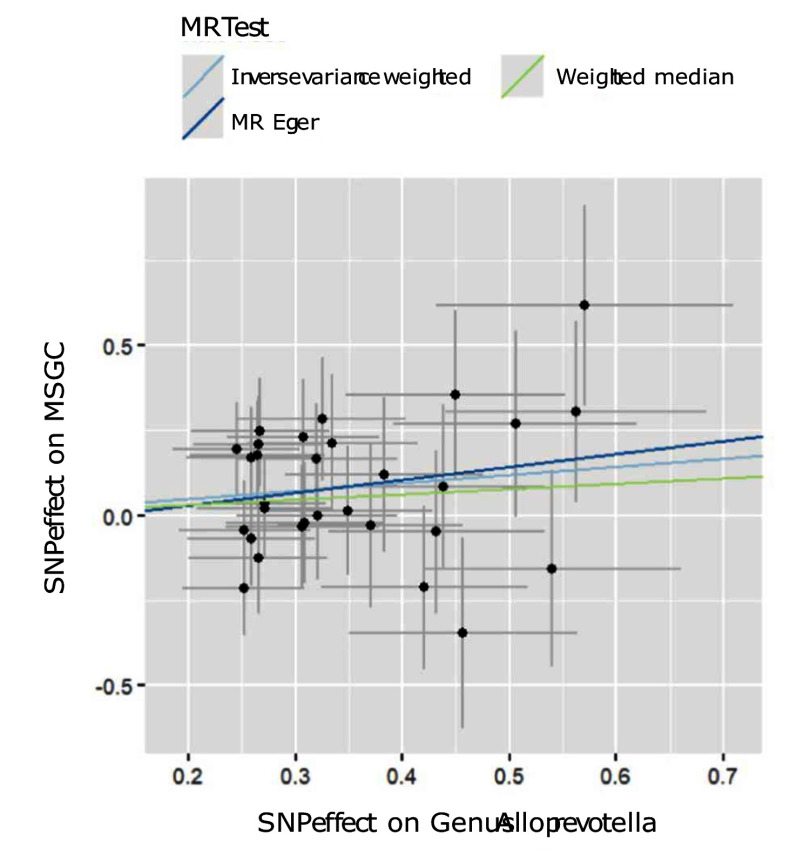
Scatter plots of three MR methods for genus *Alloprevotella* on MSGC.

**Fig 3:**
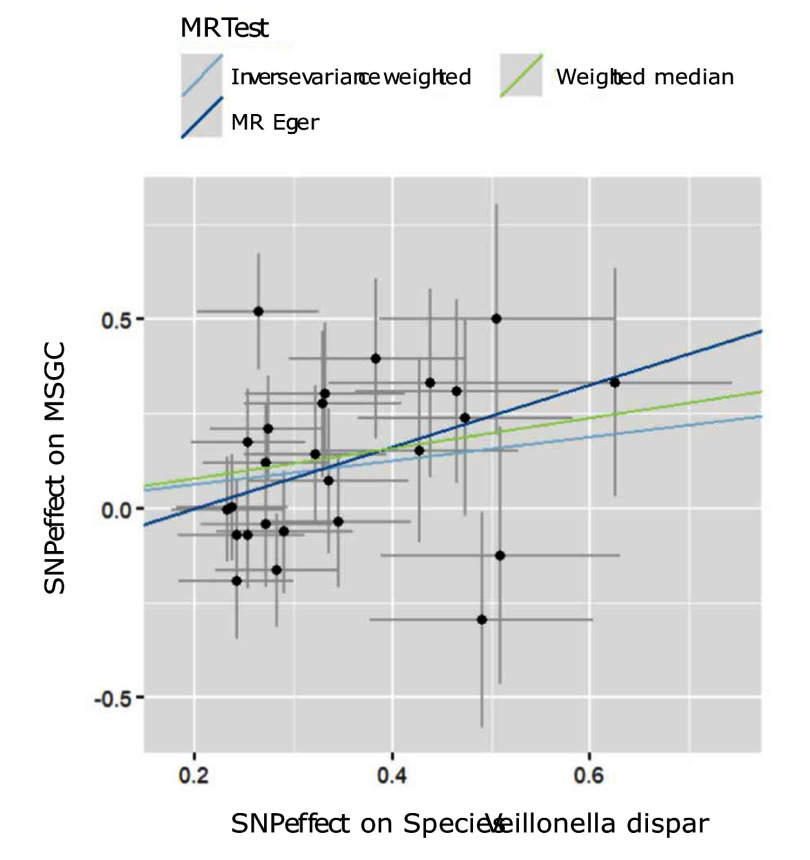
Scatter plots of three MR methods for *Veillonella dispar* on MSGC.

**Fig 4:**
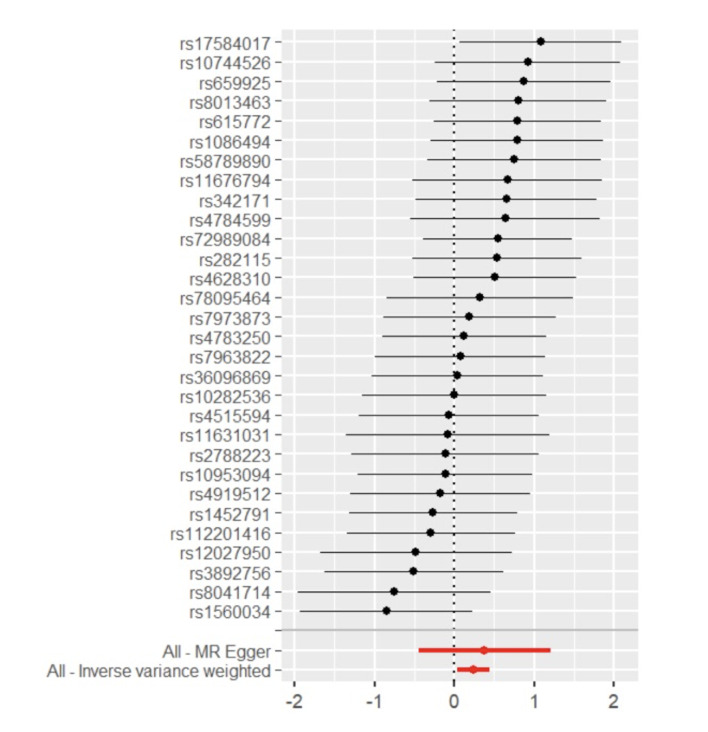
Forest plot of MR effect size for genus *Alloprevotella* on MSGC.

**Fig 5:**
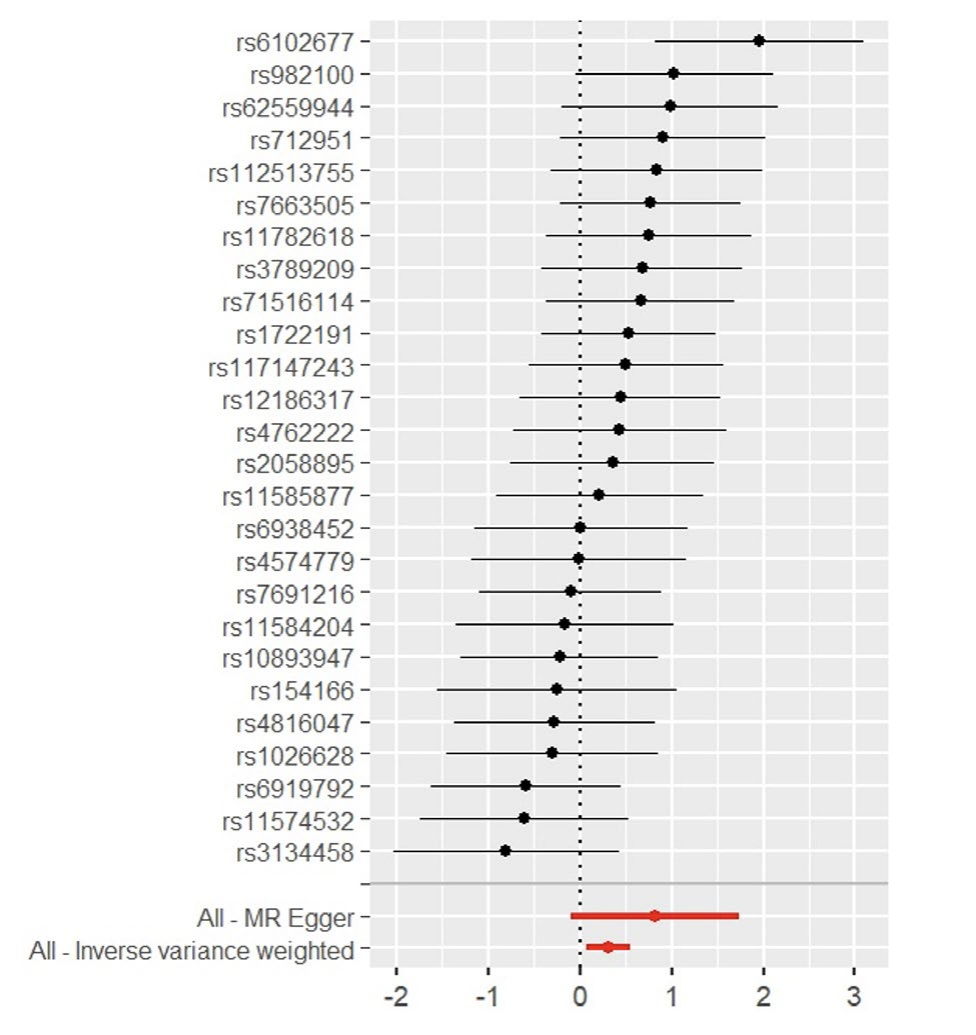
Forest plot of MR effect size for *Veillonella dispar* on MSGC.

Through the steps of IV selection mentioned above, 33 known SNPs remained, which are shown in Table 1. These SNPs were individually incorporated in the MR analysis, and the IVW method with the MSGC was used to test for statistically significant outcomes. Two kinds of SNPs, the genus *Alloprevotella* (OR = 1.267; 95%CI = 1.037-1.549; p = 0.020) and the species *Veillonella dispar* (OR = 1.369; 95%CI = 1.081-1.734; p = 0.009), were statistically significant, as shown in Figs 2 to 5 with scatter plots and forest plots. However, the MR-Egger method outcome was not statistically significant.

**Table 1:** Screening of SNPs

Exposure	Number of IVs extracted from the outcome database	SNPs excluded for being palindromic with intermediate allele frequencies	Final number of SNPs included for MR analysis
Phylum Firmicutes	50	rs17035086, rs4533614, rs919211, rs9650696	46
Phylum Proteobacteria	54	rs113400788, rs4943865, rs7171111, rs73132394, rs737476, rs9650696	48
Class Bacilli	44	rs12663639, rs1425281, rs4386119, rs494331, rs58174255	39
Order Bacteroidales	61	rs140928297, rs2322972, rs62133515, rs6852479, rs73725028, rs79977349	55
Order Fusobacteriales	38	rs11666877, rs12152661, rs1364980, rs6742182, rs9871125	33
Order Actinomycetales	46	rs1624557, rs197620, rs4375543, rs55728379, rs73384963, rs79670209	40
Order Clostridiales	60	rs10842056, rs12500446, rs17588773, rs2181142, rs291475, rs35358465, rs391729, rs732161, rs75313812, rs9832182, rs9901446	49
Family Veillonellaceae	51	rs11073493, rs35065668, rs587575, rs633370, rs72909508, rs73151073, rs7674068, rs919211, rs9650696	42
Family Pasteurellaceae	50	rs11201135, rs112822411, rs1575579, rs59739523, rs73132394, rs7974049, rs9421352	43
Family Prevotellaceae	55	rs11023461, rs16868877, rs2322972, rs6474105, rs6852479, rs6856551, rs7079192, rs7174920, rs72799473, rs79594640	44
Family Actinomycetaceae	44	rs11846904, rs4265045, rs4760559, rs503374, rs6949659, rs80293073	38
Family Lachnospiraceae	48	rs117103412, rs12500446, rs12905439, rs391729, rs9901446	43
Genus *Veillonella*	49	rs10901377, rs11603814, rs11869822, rs35065668, rs587575, rs72909508, rs73151073, rs9650696	41
Genus *Haemophilus*	48	rs10756574, rs11201135, rs112822411, rs1575579, rs34523935, rs73132394, rs76615617	41
Genus *Streptococcus*	44	rs12663639, rs5761946, rs58174255, rs73725028	40
Genus *Neisseria*	44	rs1385382, rs16955402, rs3980584, rs4894104, rs72673911	39
Genus *Prevotella*	58	rs11023461, rs112353749, rs16868877, rs2322972, rs2383049, rs2728124, rs34764917, rs635215, rs7079192, rs72799473, rs79594640	48
Genus *Porphyromonas*	43	rs138922864, rs2552135, rs28608546, rs4407994, rs71614775, rs7204511, rs74952676, rs75746897, rs9865196	34
Genus *Fusobacterium*	48	rs1018576, rs10260560, rs17355171, rs35781644, rs59123676, rs6742182, rs7520136, rs8050290, rs9526463	39
Genus *Rothia*	53	rs1357499, rs197620, rs4375543, rs4821137, rs653483, rs66497410, rs73384963, rs80113686	49
Genus *Schaalia*	63	rs17723974, rs1882273, rs197821, rs2471668, rs39543, rs4111592, rs4709179, rs6075632, rs6816828, rs6949659, rs7043159, rs73057785, rs8113541	50
Genus *Granulicatella*	44	rs10447162, rs2960744, rs55837090, rs68095140, rs72799473, rs72985394, rs78490423	37
Genus *Leptotrichia*	36	rs2712736, rs34567799, rs4912489, rs58001453, rs731880, rs7514428, rs9314525	29
Genus *Alloprevotella*	36	rs11700485, rs11736019, rs4601298, rs6135490, rs6787344	31
Species *Haemophilus parainfluenzae*	50	rs11201135, rs112822411, rs34523935, rs73132394, rs76615617, rs7974049	44
Species* Prevotella histicola*	40	rs62578379, rs72772800, rs72840580, rs76525793, rs919211	35
Species *Veillonella parvula*	40	rs10964595, rs112497666, rs12542151, rs1654451, rs3111086, rs7083373, rs73147327, rs7535113, rs9465875	31
Species *Fusobacterium periodonticum*	57	rs12916029, rs58374241, rs6067934, rs6434092, rs7204515	52
Species *Veillonella dispar*	32	rs133991, rs4716225, rs71441104, rs71668058, rs74855960	27
Species *Megasphaera micronuciformis*	47	rs1433203, rs325735, rs4241460, rs72988999	43
Species *Prevotella pallens*	41	rs10759036, rs1198063, rs12656822, rs17056583, rs2181142, rs34392760	35
Species *Rothia mucilaginosa*	38	rs292976	37
Species *Veillonella rogosae*	27	rs11029772, rs1542306, rs6980845, rs7710166	23
Phylum Firmicutes	50	rs17035086, rs4533614, rs919211, rs9650696	46
			

**Fig 6:**
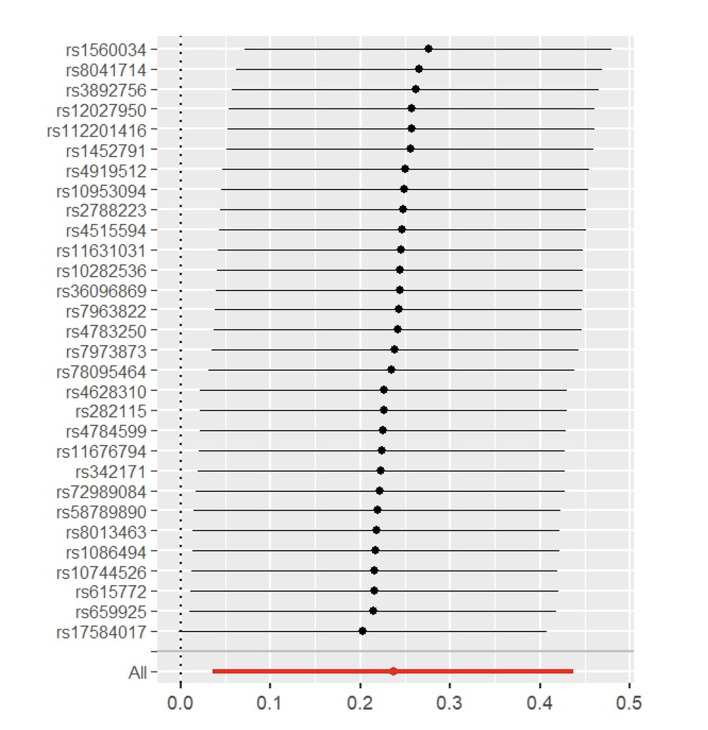
MR leave-one-out sensitivity analysis for genus *Alloprevotella* on MSGC.

**Fig 7:**
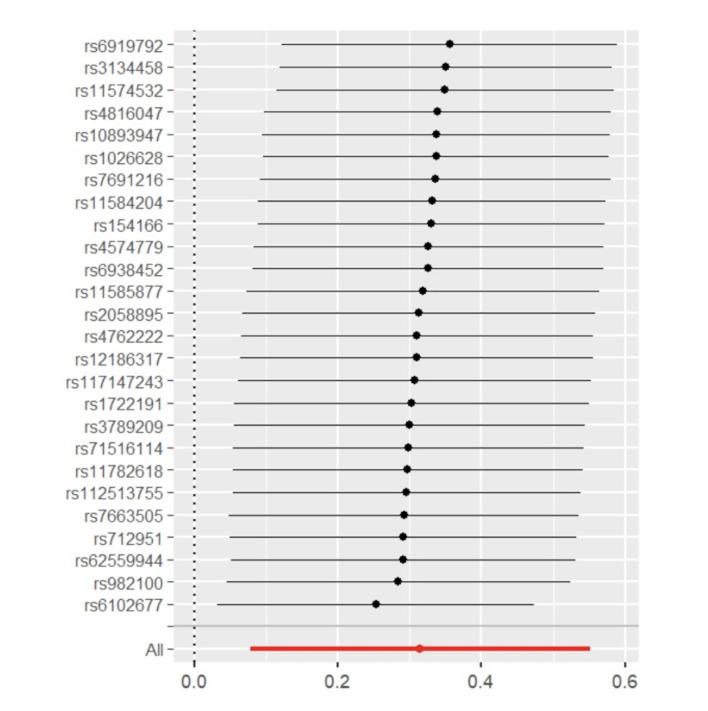
MR leave-one-out sensitivity analysis for *Veillonella dispar *on MSGC.

Subsequently, this study used Cochran’s Q test, the MR-PRESSO test, and the MR-Egger intercept test to check the robustness of these two SNPs. In Table 2, all values of the genus *Alloprevotella* (IVW: p = 0.662; MR-Egger: p = 0.618) and the species *Veillonella dispar* (IVW: p = 0.232; MR-Egger: p = 0.246) were greater than 0.05, which suggests that there was no heterogeneity in the results. In the MR-PRESSO test, no abnormal values were detected. In the columns of the MR-Egger intercept test, both the values of the genus *Alloprevotella* (p = 0.731) and the species *Veillonella dispar* (p = 0.277) were greater than 0.05, indicating that the results contained no pleiotropy. Finally, the leave-one-out sensitivity analysis results, shown in Figs 6 and 7, revealed that removing any single SNP did not statistically significantly affect the positive outcomes, indicating the robustness of the present results.

**Table 2:** Causal estimations of the effects of the oral microbiota on MSGC in the MR analysis

Exposure	Outcome
SNPs	MR Model	OR	95%Cl	p	Cochran’s Q test	MR-Egger intercept test
Genus *Alloprevotella*	31	IVW MR-Egger	1.267 1.460	1.037-1.549 0.641-3.323	0.020 0.375	0.662 0.618	0.731 0.277
Species *Veillonella dispar*	27	IVW MR-Egger	1.369 2.268	1.081-1.734 0.904-5.690	0.009 0.094	0.232 0.246


### Reverse MR Analysis Results

**Fig 8:**
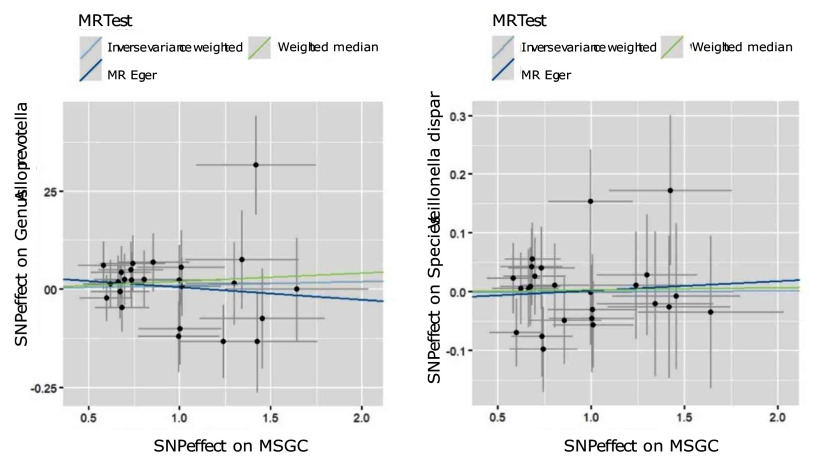
Scatter plots of reverse causal relationships.

**Fig 9:**
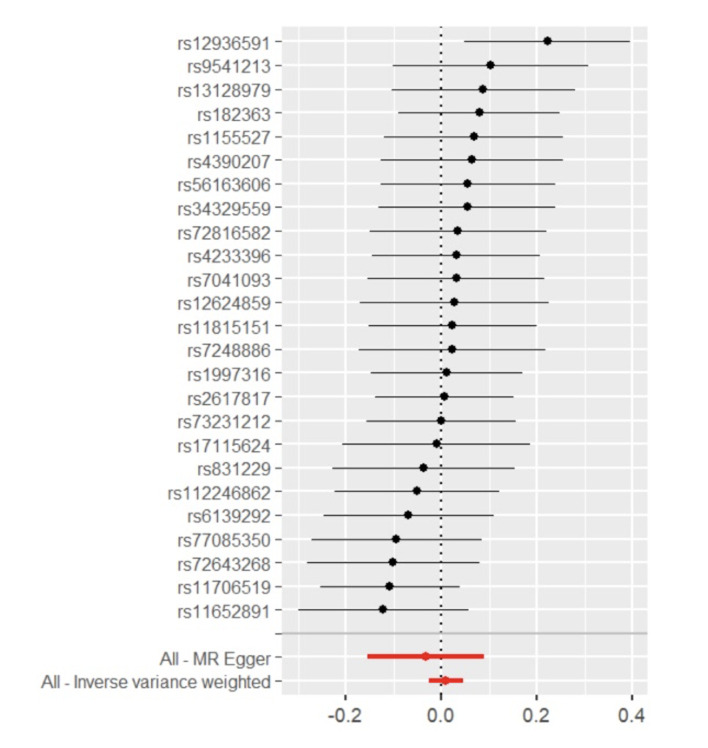
Forest plots of reverse causal relationships for MSGC on genus *Alloprevotella*.

**Fig 10:**
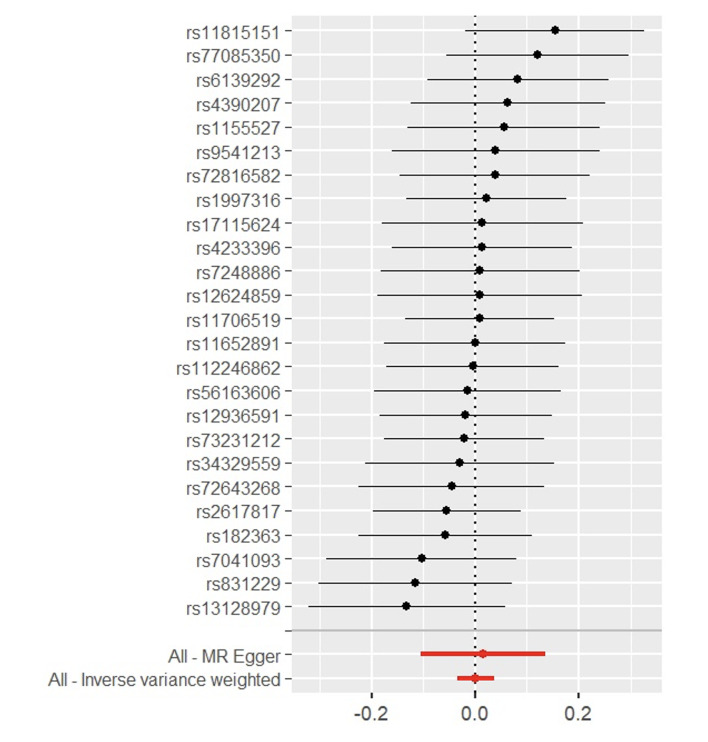
Forest plots of reverse causal relationships for MSGC on *Veillonella dispar*.

To deny the potential reverse causal relationship, MR analysis was performed again by using the MSGC as the exposure variable and the genus *Alloprevotella* and the species *Veillonella dispar* as outcome variables. The results are presented in Table 3. IVW analysis revealed that both the genus *Alloprevotella* (OR = 1.010; 95%CI = 0.974-1.046; p = 0.596) and the species *Veillonella dispar* (OR = 1.000; 95%CI = 0.967-1.036; p = 0.957) were not statistically significantly associated with MSGC. Similarly, no statistically significant effects on MSGC were detected for the genus *Alloprevotella* (OR = 0.969; 95%CI = 0.857-1.095; p = 0.614) or the species *Veillonella dispar* (OR = 1.016; 95%CI = 0.901-1.145; p = 0.798) via MR-Egger analysis. The scatter plots and forest plots are presented in Figs 8 to 10. In addition, as in the previous section, the Cochran’s Q test, MR-PRESSO test, and MR-Egger intercept test were used to examine robustness; the results also support a lack of reverse causal relationship between MSGC and the genus *Alloprevotella* as well as the species *Veillonella dispar*.

**Table 3:** Examination of reverse causal relationships via MR analysis

Exposure-outcome	Reverse MR analysis result
SNPs	MR Model	OR	95%Cl	p	Cochran’s Q test	MR-Egger intercept test
MSGC-*Alloprevotella*	35	IVW	1.010	0.974 -1.046	0.596	0.802	0.494 0.802
MSGC-*Veillonella dispar*	31	MR-Egger IVW MR-Egger	0.969 1.000 1.016	0.857-1.095 0.967-1.036 0.901-1.145	0.614 0.957 0.798	0.782 0.966 0.953


## DISCUSSION

The oral microbiota, one of the most complex microbial communities in the human body, has been shown to significantly influence the occurrence of systemic cancers, i.e., malignant tumors that occur in organs outside the oral cavity (such as colorectal cancer, pancreatic cancer, etc). Among them, specific bacteria, such as *Streptococcus, Peptostreptococcus, Prevotella, Porphyromonas gingivalis*, and *Fusobacterium nucleatum*, are closely associated with the development of oral squamous cell carcinoma.^47^ Studies have shown that the influence of oral microbiota is not limited to the oral cavity. For example, *Fusobacterium nucleatum* and *Porphyromonas gingivalis* are significantly associated with the development of colorectal cancer and pancreatic cancer, play a key role in their development. The mechanisms may involve bacterial translocation, inflammation induction, and immune regulation.^13,32^

The genus *Alloprevotella*, a member of the family Prevotellaceae, has been shown to produce short-chain fatty acids in the gut, contributing positively to maintaining normal immune homeostasis.^49^ However, under unhealthy conditions, it has the potential to act as an opportunistic pathogen. Additionally, previous studies have linked the genus *Alloprevotella* to various extraoral diseases, such as autism^31^ and rheumatoid arthritis.^48^ Studies have also revealed a positive correlation between the abundance of the genus *Alloprevotella* and various extraoral cancers, such as gastrointestinal malignancies^30^ and cholangiocarcinoma.^33^ Additionally, studies have identified this genus as a risk factor for the development of oral cancer via the Mendelian randomization method.^20^ A study on changes in the gut microbiota of HIV patients revealed that the abundance of *Alloprevotella* was statistically significantly greater in HIV-positive individuals than in healthy controls, suggesting a potential association with infection.^21^ Another similar study revealed that the abundance of *Alloprevotella* was positively correlated with the number of CD4+ T-cells.^29^ These findings indicate that *Alloprevotella* may be closely linked to the body’s immune response, with its abundance potentially playing a role in activating the immune system. Regarding the mechanism of activating the immune system, a considerable number of studies believe that the flora affects the immune system by regulating the activity of immune checkpoints.^44^ Immune checkpoints refer to a category of molecular mechanisms within the immune system that regulate the intensity of immune responses.^15^ They primarily function through co-inhibitory receptors (such as PD-1 and CTLA-4) expressed on the surface of T-cells. These receptors bind to their corresponding ligands, transmitting inhibitory signals that prevent excessive T-cell activation and the onset of autoimmune reactions. In cancer, tumor cells upregulate these immune checkpoint molecules to evade recognition and attack by the immune system, thereby promoting tumor growth and metastasis. Immune checkpoint inhibitors (ICIs) are a class of monoclonal antibodies designed to block these inhibitory pathways, restore the tumor-killing function of T-cells, and reactivate anti-tumor immune responses.

Studies have shown that bacteria and the gut microbiota influence the efficacy of immune checkpoints and their inhibitors through multiple mechanisms.^34^ On the one hand, specific beneficial bacteria (such as *Bacteroides fragilis, Akkermansia muciniphila,* and *Bifidobacterium*) can enhance the effectiveness of ICIs by activating dendritic cells, promoting the secretion of cytokines such as IL-12, and increasing tumor infiltration and cytotoxicity of CD8+ T-cells.^41^ On the other hand, microbial metabolites, including short-chain fatty acids and inosine, can also potentiate anti-tumor immunity by modulating immune cell functions and signaling pathways, such as the STING–IFN-I pathway.^22,24^ Furthermore, alterations in the composition of the microbiota (e.g., antibiotic-induced dysbiosis) may lead to an increase in immunosuppressive cells (such as MDSCs or Tregs) or reduce the responsiveness of immune cells to ICIs, thereby affecting treatment outcomes.^34^

*Veillonella dispar* is a relatively rare Gram-negative anaerobic coccus and a member of the normal microbiota in the oral cavity and gastrointestinal tract. It is generally nonpathogenic. However, owing to its strong antibiotic resistance, it may act as an opportunistic pathogen under disease conditions.^42^ Previous studies have reported that it can cause bacteremia in cancer patients.^3,12^ Furthermore, multiple studies suggest that changes in the abundance of *Veillonella dispar* could serve as potential biomarkers for various cancers, including lung adenocarcinoma, hepatocellular carcinoma, nasopharyngeal carcinoma, and pancreatic ductal adenocarcinoma,^8,11,25,35^ indicating a potential correlation with cancer development.

This study, which used Mendelian randomization, identified a positive causal relationship between *Alloprevotella* and *Veillonella dispar* and MSGC, establishing both as risk factors for major salivary gland cancer. Combining these findings with those of previous studies, it is reasonable to assert that both bacteria are associated with immune dysfunction and the development of various tumors. These findings suggest that these bacteria may promote cancer by triggering infections or immune responses that alter the intracellular environment. Alternatively, their impact may involve other mediating factors within the body, contributing to the onset of MSGC. Notably, Fusobacterium and Porphyromonas, which are established risk factors for periodontal disease and oral cancer,^46^ did not exhibit a statistically significant impact on MSGC in this study, nor did they show a statistically significant causal relationship with MSGC. This observation may be attributed to the distinct biological features of salivary gland cancer:

The anatomical location within the salivary duct system limits direct microbial exposure. Fusobacterium nucleatum’s two main virulence factors are fusobacterial adhesin A and Fap2, which are derived from Fusobacterium nucleatum. The former is an important kinase in oral squamous cell carcinoma, and the latter is a substance that interacts with the T-cell immune receptor with Ig and ITIM domains to inhibit the activity of immune cells. Both require direct contact with the diseased area to exert their maximum effect.
Differences in the tumor microenvironment, such as low oxygen tension, may impair microbial survival.^19,45^

Further clinical evidence and mechanistic studies are needed to clarify their role. Additionally, this study has certain limitations. First, it was based on a GWAS database and focused on genus/species-level microbial classification. However, we should consider the potential for differences in pathogenicity among strains within the same genus. Future studies combining metagenomic sequencing to elucidate strain-specific effects could further complement this finding. Furthermore, due to database limitations, these findings may not be generalizable to non-European populations.

## CONCLUSION

This study revealed a positive causal relationship between the genus *Alloprevotella* and the species *Veillonella dispar* with major salivary gland cancer (MSGC), establishing them as risk factors for MSGC. This finding highlights the potential role of microbiological factors in MSGC development and provides valuable insights for further exploration of its underlying mechanisms.
